# Life expectancy of HIV-positive patients after diagnosis in Iran from 1986 to 2016: A retrospective cohort study at national and sub-national levels

**DOI:** 10.4178/epih.e2018053

**Published:** 2018-11-07

**Authors:** Mohammad Mirzaei, Maryam Farhadian, Jalal Poorolajal, Parvin Afasr Kazerooni, Katayoun Tayeri, Younes Mohammadi

**Affiliations:** 1 Department of Epidemiology, School of Public Health, Hamadan University of Medical Sciences, Hamadan, Iran; 2 Department of Biostatistics, School of Public Health, Hamadan University of Medical Sciences, Hamadan, Iran; 3 Modeling of Noncommunicable Diseases Research Center, Department of Biostatistics and Epidemiology, School of Public Health, Hamadan University of Medical Sciences, Hamadan, Iran; 4 HIV/AIDS Research Centre, Shiraz University of Medical Sciences, Shiraz, Iran; 5 Iranian Research Center of HIV & AIDS, Tehran University of Medical Sciences, Tehran, Iran; 6 Social Determinants of Health Research Center, School of Public Health, Hamadan University of Medical Sciences, Hamadan, Iran

**Keywords:** Life expectancy, Human immunodeficiency virus, Tuberculosis, Iran

## Abstract

**OBJECTIVES:**

Little is known about the life expectancy of individuals with human immunodeficiency virus (HIV) in Iran. This study therefore aimed to estimate the life expectancy of HIV-positive patients in Iran.

**METHODS:**

In this retrospective cohort study, we extracted data from the Center for Disease Control and Prevention of the Ministry of Health and Medical Education and the Death Registration System. We included patients aged 20 years and older who had a specified date of diagnosis. We estimated life expectancy and its 95% confidence intervals (CIs) using Chiang’s methodology.

**RESULTS:**

The overall life expectancy at the national level was 23.1 years (95% CI, 22.6 to 23.5). Life expectancy was 21.6 years (95% CI, 21.1 to 22.0) for men and 32.7 years (95% CI, 31.4 to 34.0) for women. The life expectancy of patients who did or did not receive antiretroviral therapy (ART) was 37.0 years (95% CI, 36.2 to 37.8) and 15.5 years (95% CI, 15.1 to 15.9), respectively. The life expectancy of patients with or without tuberculosis (TB) was 21.6 years (95% CI, 20.4 to 22.9) and 36.5 years (95% CI, 35.7 to 37.4), respectively.

**CONCLUSIONS:**

The life expectancy of Iranian HIV-positive patients was found to be very low. To improve their longevity, improvements in ART coverage and the control and treatment of TB are advised.

## INTRODUCTION

Since 1981, human immunodeficiency virus (HIV) and acquired immune deficiency syndrome (AIDS) have remained among the most important public health challenges worldwide [1,2]. Annually, 2.1 million people are infected with HIV and 1.1 million die of AIDS [3]. Accordingly, by the end of 2015, approximately 37 million people globally were living with HIV [3].

In Iran, the first case of HIV was diagnosed in 1986 following a blood transfusion in a girl with hemophilia. Combined antiretroviral therapy (ART) was introduced in 1997 [4,5]. Following the first case, the number of reported new cases of HIV increased slowly, and in 1996 the occurrence of HIV was first reported among prisoners [5]. Based on the latest reports, through 2016, more than 32,000 cases of HIV had been registered with the Ministry of Health and Medical Education (MOHME), although estimates have suggested that the number of HIV-positive individuals in the Iranian population may exceed 75,000 [5,6]. Although a previous report stated that most cases of HIV occur among people who inject drugs (PWID), trends in transmission routes indicate that HIV is increasingly spread through sexual relations. However, according to the evidence, the HIV epidemic in Iran can be described as concentrated among PWID [5].

Based on statements made at 3 meetings in 2003, 2008, and 2011, Iran has committed itself to taking measures to control the AIDS epidemic and to provide health and medical services for affected patients [5]. However, no study has yet investigated the effectiveness of health and medical interventions for AIDS patients or monitored the health status of HIV/AIDS patients in Iran. Life expectancy is a useful indicator that may help policy-makers assess the health status of the general population [7,8]. Life expectancy at a specific age is defined as the average number of years that a person expects to survive beyond that age [9-11]. This indicator reflects individuals’ health status and can be used to assess the effectiveness of plans and interventions [7-9,11]. Although many studies have found AIDS to have a significant effect on longevity in various communities [9,12-14], the available information on the health status of HIV/AIDS patients in Iran, including life expectancy, is not very clear. Therefore, to increase our knowledge of the effect of AIDS on life expectancy in Iranian patients, and to assess the effectiveness of antiretroviral interventions, we need to obtain this information. In this study, we aimed to estimate the life expectancy of Iranian HIV-positive patients from 1987 to 2016 at both the national and sub-national levels.

## MATERIALS AND METHODS

### Population and data sources

In this study, our population data included all Iranian individuals aged 20 years and over with a confirmed HIV test who had been registered with the Center for Disease Control and Prevention (CDC) of the MOHME. The CDC is responsible for education, surveillance, identification, examination, registration, follow-up, and treatment of HIV/AIDS patients. It registers demographic information, such as age, gender, residential area, job, and marital status, as well as clinical characteristics, such as the date of diagnosis and the results of primary screening for the disease, including tuberculosis (TB) status and the CD4 count. In this study, we extracted information on the following parameters from the database: age, gender, province, date of diagnosis, date of last health care services, stage of disease, date of death or censoring, and CD4 cell count.

We obtained death data from the Death Registration System (DRS) of the MOHME, which is linked with the CDC. The role of this system is to record cases of death with information such as age, gender, cause of death, date of death, place of residence, and place of death.

To calculate life expectancy, we included patients who had a date of diagnosis that preceded their death. Therefore, we excluded patients with no date of diagnosis, or with a date of diagnosis that coincided with death or was registered after death. Within those limitations, all HIV-positive individuals aged 20 years and older from all parts of Iran were included in the study.

### Statistical analysis

Before calculating life expectancy, we imputed missing values for age at diagnosis and gender. We imputed these variables, using the Amelia package in R, because they were missing in fewer than 10% of cases. We used Chiang’s methodology to construct a bridge life table with which to calculate life expectancy [15]. More specifically, we constructed a life table based on 5-year age groups from 20 to 65 years and older. Then, for each age group, we obtained the sum of person-years lived by that age group and the number of deaths from the DRS. To obtain the person-years, we subtracted the time of death, the time of censorship, or the time when the study finished. We calculated the age-specific mortality rate by dividing the number of deaths by the sum of person-years. These age-specific mortality rates were converted into the probability of dying for each age group. In the next step, we obtained the sum of person-years lived by every specific age-group. Then, we obtained the number of person-years lived by a cohort above the exact age x, and finally, by dividing the number of person-years lived by the number of members of the cohort at the exact age x, life expectancy was calculated. We calculated life expectancy by age (categorized into 5-year groups from 20 to 64 and ≥65 years), gender, CD4 cell count (less than 200 and more than 200), TB, the 4 clinical stages defined by the World Health Organization (WHO), ART, marital status, route of transmission, and province of residence in Iran. We constructed the lower and upper limits of life expectancy by multiplying the standard error by 1.96. The variance of life expectancy was obtained as follows:

(1) $$S_{ei}^{2}=\frac{1}{{l^{2}}_{i}}\sum_{j=i}^{w=1}{l^{2}}_{j}\left[\left(1-a_{j}\right)n_{j}+e_{j+1}\right]{S^{2}}_{Pj}$$

Where the variance of quantity *p* is:

(2) $${S^{2}}_{P_{j}}=\frac{{q^{2}}_{j}p_{j}}{D_{j}}$$

where, i=1,….10, *e_i_* is the life expectancy at age *i*, *l_i_* is the number of alive people at the start of age interval *i*, *a_i_* is the fraction of last age interval survived, and n is the age interval width.

We used Microsoft Excel version 2010 (Microsoft, Redmond, WA, USA) and R version 3.2.1 (https://cran.r-project.org/bin/windows/base/old/3.2.1/) to run the analysis. The p-values less than 0.05 were considered to indicate statistical significance. We conducted this study in accordance with the GATHER (Guidelines for Accurate and Transparent Health Estimates Reporting) statement [16].

## RESULTS

Table 1 shows the demographic characteristics of Iranian HIV/AIDS patients from 1987 through December 30, 2016. Of the 30,516 HIV-positive patients, 25,850 (84.7%) were aged 20-44 years and 25,507 (83.6%) were men. Furthermore, 10,138 (33.2%) patients were married and 10,676 (35.0%) were single. Injection drug use was the most common route of transmission (66.9%). The prevalence of TB infection among the patients was 3.7%.

Table 2 shows the estimated life expectancy of HIV/AIDS patients. The total duration of follow-up for the included patients was 96,924 person-years, with a median follow-up of 3.4 years per patient. The overall crude mortality rate (CMR) of the patients was 37.2 per 1,000 person-years, and the CMR of men (41.7 per 1,000 person-years) was higher than that of women (15.9 per 1,000 person-years). A total of 97,528 years of life were lost among HIV/AIDS patients from 1987 to 2016. Our analysis showed that the life expectancy of Iranian HIV-positive patients at 20 years of age was 23.1 years (95% confidence interval [CI], 22.6 to 23.5) overall, 21.6 years (95% CI, 21.1 to 22.0) for men, and 32.7 years (95% CI, 31.4 to 34.0) for women.

**Figure 1.**
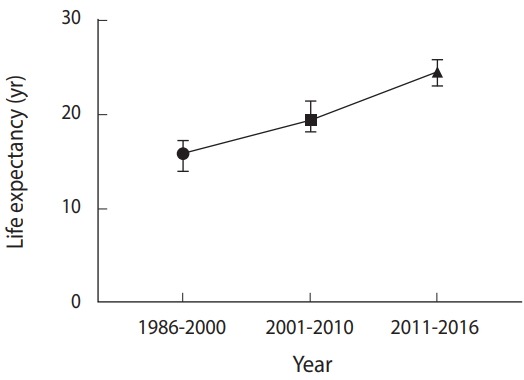
Time trend of life expectancy of Iranian HIV/AIDS patients.

Life expectancy increased from 15.8 years in the period of 1986 to 2000 to 24.6 years in 2016 (Figure 1).

In addition, the life expectancy of patients who received ART was 37.0 years (95% CI, 36.2 to 37.8), while in those who did not receive ART, it was 15.5 years (95% CI, 15.1 to 15.9; p<0.001). As shown in Table 2, patients who had a CD4 count ≥200 cells/mm^3^ at the time of diagnosis had a life expectancy of 32.8 years (95% CI, 30.8 to 34.7), while that of those who had a CD4 count <200 cells/mm^3^ at diagnosis was 17.8 years (95% CI, 15.5 to 20.2; p<0.001). As shown in Table 2, the life expectancy of individuals with a co-infection of HIV and TB was 21.6 years (95% CI, 20.4 to 22.9), while the life expectancy of HIV patients without TB was 36.5 years (95% CI, 35.7 to 37.4; p<0.001). A significant difference in life expectancy was found according to the clinical stage of HIV. Stages I and IV had the highest and the lowest life expectancy, respectively. The mother-to-child transmission route was associated with the lowest life expectancy, at 10.7 years (95% CI, 10.2 to 11.2), while sexual transmission was associated with the highest life expectancy, at 31.4 years (95% CI, 30.3 to 32.4) (Table 2). In addition, life expectancy in married patients was higher than in single patients (25.5 vs. 20.3 years, respectively).

Table 3 presents the results of life expectancy at the sub-national level. Major differences were found across the provinces of Iran, with life expectancy ranging from 13.2 years (95% CI, 11.6 to 14.8) in Kohgiluyeh and Boyer-Ahmad Province to 31.4 years (95% CI, 30.2 to 32.6) in Tehran.

## DISCUSSION

In this study, our aim was to estimate the life expectancy of HIV/AIDS patients in Iran. The literature indicates that life expectancy has improved among the Iranian general population [17,18]. Statistics have demonstrated that the life expectancy of the Iranian people increased from 67 years in 1990 to over 75 years in 2015 [19]. Despite these dramatic advances in the general population, our results indicated a low life expectancy in HIV-positive patients in Iran. The results showed a 33-year gap between the life expectancy of the general population and that of the HIV-infected population, which indicates the impressive effects of AIDS on life expectancy. In addition, sub-group analyses showed differences in life expectancy by gender, ART, CD4 count, TB, transmission route, disease stage, and province. In terms of the effect of gender, the life expectancy of women is higher than that of men even in the general population. However, a part of this discrepancy in the life expectancy between women and men can be attributed to the effect of ART. The results showed that the percentage of patients taking ART was higher among women than among men. However, the largest differences in life expectancy were due to ART and TB. Patients who received ART lived 22 years longer than those patients who did not. Moreover, the gap between patients with and without TB was about 15 years. In this study, we found that two of the most important factors affecting life expectancy were ART and TB. Therefore, it seems that providing ART and controlling TB can play an important role in improving the life expectancy of HIV patients. Several studies have confirmed the effectiveness of ART on life expectancy and the survival rate [9,20,21].

At the sub-national level, we found a massive gap in life expectancy among provinces. The difference between the highest and the lowest life expectancy was more than 18 years. We attempted to find explanations for this disparity. Generally, the provinces with the lowest life expectancy share several features. They are socially and economically deprived provinces located in border regions that have a lower Human Development Index than other provinces [22]. These provinces also have the lowest life expectancy in the general population. Our findings revealed that the coverage of ART in the provinces with lowest life expectancy was very low and the incidence of TB was high [23].

Moreover, we examined non-health factors in the provinces. We found a relatively strong correlation of life expectancy with educational level (r=0.64) and the wealth index (r=0.61). In general, the provinces with a high life expectancy had a high educational level and high wealth index and vice-versa. Therefore, based on our results, we recommend that policy-makers should consider these factors, especially ART, to improve life expectancy in HIV patients. We hope that consistent ART use will allow HIV patients to have an almost normal life expectancy, similar to that of the general population.

This study was affected by several limitations. The first limitation was the under-registration of AIDS patients and death events. Despite the presence of a system to identify and treat patients, estimates have shown that this system does not detect all patients. Based on the latest estimates in Iran, the number of HIV patients is estimated to be over 75,000, while the system has identified just 32,000 [5,6]. To determine a precise estimate of the life expectancy of HIV/AIDS patients, we need information on all patients. In addition to the under-registration of AIDS patients, the Iranian DRS faces problems related to under-registration and misclassification [24]. These patterns of under-registration may have affected our results, making it necessary to interpret them cautiously. Second, we excluded many patients (5.4%) from the study because we did not have enough information on their follow-up. If the life expectancy of the censored patients is not the same as those who were included in the study, our results might be biased. An analysis based on demographic characteristics, however, did not show any significant differences between these groups. Third, at the national level, due to the non-registration of patients in newly-formed provinces such as South Khorasan and North Khorasan, we could not estimate the life expectancy for those provinces separately; therefore, we incorporated the corresponding findings into a single province.

In conclusion, the life expectancy of Iranian HIV-positive patients is much lower than that of the general population, and there are wide gaps across sub-groups of the HIV-positive population by province, gender, ART, and TB. Iranian health policy-makers need to consider these factors. In particular, increasing the coverage of ART and controlling TB should improve the survival of HIV patients. In combination with measures such as early detection, such initiatives will help Iran achieve the objectives of the 90-90-90 program of the WHO by 2020.

## Figures and Tables

**Table 1.** Baseline and clinical characteristics of HIV-positive patients in Iran (1987-2016)

Characteristics	n (%)
Gender	
Men	22,507 (83.6)
Women	5,009 (16.4)
Age (yr)	
0-4	349 (1.1)
5-14	175 (0.5)
15-24	3,188 (10.4)
25-34	14,089 (46.1)
35-44	9,076 (29.7)
45-54	2,937 (9.6)
55-64	593 (1.9)
≥65	109 (0.3)
Marriage status	
Married	10,138 (33.2)
Single	10,676 (34.9)
Divorced or widowed	4,941 (16.1)
Unknown	4,761 (15.6)
Transmission	
IDU	20,415 (66.9)
Unsafe sexual contact	5,767 (18.9)
Blood transfusion	214 (0.7)
Mother-to-child	488 (1.6)
Unknown	3,661 (12.0)
WHO disease stage	
I	8,046 (57.9)
II	2,323 (16.0)
III	2,305 (16.6)
IV	1,293 (9.3)
Co-infection of HIV and TB	
Positive	1,136 (3.7)
Negative	10,659 (34.9)
Unknown	18,721 (61.3)
CD4 cell count at baseline (cells/mm^3^)	
≤200	1,658 (37.6)
201-349	891 (20.2)
350-499	731 (16.6)
≥500	1,126 (25.5)
Antiretroviral therapy	
Yes	10,288 (33.7)
No	20,228 (66.2)
Prognosis	
Alive	9,613 (31.5)
Censored	12,708 (41.6)
Died	8,195 (26.8)

HIV, human immunodeficiency virus; IDU, injection drug use; WHO, World Health Organization; TB, tuberculosis.

**Table 2.** Life expectancy among HIV-positive patients by demographic and clinical variables (1987-2016)

Variables	20-24	25-29	30-34	35-39	40-44	45-49	50-54	55-59	60-64	≥65
Overall life expectancy in Iran (2015) [17]	56.1	51.5	46.8	42.1	37.3	32.6	28.0	23.6	19.3	15.2
Overall in HIV/AIDS patients	23.1 (22.6, 23.5)	19.7 (19.3, 20.0)	16.9 (16.5, 17.3)	14.5 (14.1, 14.9)	12.6 (12.1, 13.0)	10.7 (10.2, 11.2)	8.9 (8.4, 9.5)	7.5 (7.0, 8.1)	5.5 (5.0, 6.0)	2.5 (2.2, 2.7)
Men	21.6 (21.1, 22.0)	18.4 (18.0, 18.8)	15.8 (15.4, 16.2)	13.5 (13.1, 13.9)	11.7 (11.2, 12.1)	9.9 (9.3, 10.4)	8.2 (7.6, 8.8)	6.8 (6.1, 7.4)	5.3 (4.7, 6.0)	2.5 (2.1, 2.8)
Women	32.7 (31.4, 34.0)	28.3 (27.1, 29.6)	24.6 (23.4, 25.8)	20.9 (19.6, 22.1)	17.6 (16.3, 18.7)	14.6 (13.4, 15.9)	11.2 (10.0, 12.5)	9.6 (8.5, 10.7)	6.0 (5.0, 6.9)	2.5 (2.2, 2.7)
Marriage status										
Married	25.5 (24.8, 26.2)	21.5 (20.9, 22.1)	17.9 (17.4, 18.5)	15.2 (14.6, 15.80)	12.9 (12.2, 13.5)	10.5 (9.8, 11.1)	8.8 (8.1, 9.6)	7.3 (6.5, 8.1)	5.4 (4.7, 6.2)	2.5 (2.1, 2.9)
Single	20.3 (19.6, 20.9)	17.0 (16.4, 17.6)	14.5 (13.8, 15.1)	12.2 (11.5, 12.9)	10.4 (9.5, 11.2)	9.3 (8.2, 10.4)	7.2 (5.9, 8.5)	5.2 (3.5, 6.8)	4.2 (3.0, 5.3)	2.5 (2.2, 2.7)
Divorced or widowed	27.9 (26.9, 29.0)	23.7 (22.8, 24.6)	20.3 (19.4, 21.2)	17.1 (16.2, 18.0)	14.9 (13.9, 15.8)	12.8 (11.7, 13.8)	10.8 (9.7, 11.9)	9.5 (8.5, 10.5)	6.2 (5.5, 7.0)	2.5 (2.1, 2.8)
Unknown	13.1 (11.8, 14.5)	10.5 (9.5, 11.5)	9.0 (8.0, 9.9)	8.2 (7.2, 9.2)	7.6 (6.5, 8.7)	6.3 (5.3, 7.4)	3.8 (2.9, 4.7)	3.0 (2.7, 3.4)	2.8 (2.4, 3.1)	2.5 (2.2, 2.7)
WHO disease stage										
I	38.1 (37.1, 39.1)	33.7 (32.8, 34.7)	29.8 (28.8, 30.8)	25.6 (24.6, 26.5)	21.9 (20.9, 22.9)	18.4 (17.4, 19.4)	14.7 (13.7, 15.7)	11.1 (10.2, 12.0)	7.0 (6.2, 7.7)	2.5 (1.9, 3.0)
II	37.7 (35.9, 39.5)	33.3 (31.5, 35.0)	28.6 (26.8, 30.4)	24.5 (22.7, 26.2)	20.7 (18.9, 22.5)	17.1 (15.3, 19.0)	13.8 (11.9, 15.6)	10.2 (8.6, 11.9)	6.8 (5.4, 8.2)	2.5 (1.6, 3.3)
III	24.6 (23.4, 25.8)	20.4 (19.3, 21.5)	17.3 (16.2, 18.4)	14.4 (13.3, 15.5)	12.1 (10.9, 13.3)	9.8 (8.5, 11.1)	8.1 (6.6, 9.6)	8.0 (6.3, 9.6)	5.7 (4.1, 7.2)	2.5 (1.7, 3.2)
IV	24.8 (23.3, 26.4)	20.5 (19.1, 21.8)	16.8 (15.4, 18.2)	14.1 (12.7, 15.5)	11.6 (10.2, 13.1)	9.0 (7.4, 10.7)	7.3 (5.5, 9.2)	6.7 (4.6, 8.8)	6.1 (4.6, 7.6)	2.5 (1.4, 3.5)
Co-infection of HIV and TB										
TB-positive	21.6 (20.4, 22.9)	17.1 (16.0, 18.3)	14.1 (13.0, 15.2)	11.5 (10.4, 12.5)	9.5 (8.4, 10.5)	6.7 (5.6, 7.7)	3.4 (2.3, 4.6)	4.5 (2.5, 6.5)	3.7 (1.6,5.8)	2.5 (1.8, 3.1)
TB-negative	36.5 (35.7, 37.4)	32.1 (31.3, 32.9)	27.9 (27.1, 28.7)	23.9 (23.1, 24.7)	20.4 (19.4, 21.3)	16.9 (16.1, 17.8)	13.8 (13.0, 14.7)	10.6 (9.8, 11.4)	7.0 (6.3, 7.6)	2.5 (1.5, 3.0)
CD4 count at baseline (cells/mm^3^)										
≤200	17.8 (15.5, 20.2)	15.2 (13.6, 16.8)	14.5 (13.2, 15.7)	12.7 (11.4, 14.0)	11.5 (10.1, 12.9)	10.5 (9.0, 12.0)	8.5 (6.9, 10.1)	9.0 (7.4, 10.5)	5.8 (4.4, 7.2)	2.5 (1.7, 3.2)
>200	32.8 (30.8, 34.7)	29.0 (27.2, 30.9)	25.5 (23.7, 27.4)	21.6 (19.8, 23.5)	18.2 (16.3, 20.1)	15.6 (13.7, 17.5)	12.4 (10.5, 14.3)	10.0 (8.3, 11.6)	6.4 (5.0, 7.9)	2.5 (1.6, 3.4)
Antiretroviral therapy										
Yes	37.0 (36.2, 37.8)	32.4 (31.6, 33.2)	28.0 (27.2, 28.8)	23.9 (23.1, 24.6)	20.3 (19.5, 21.1)	16.6 (15.8, 17.5)	13.2 (12.4, 14.0)	10.5 (9.7, 11.2)	6.6 (5.9, 7.2)	2.5 (2.1, 2.9)
No	15.5 (15.1, 15.9)	12.5 (12.2, 12.8)	10.4 (10.1, 10.7)	8.8 (8.5, 9.1)	7.5 (7.2, 7.9)	6.3 (5.9, 6.6)	4.8 (4.4, 5.3)	3.9 (3.3, 4.4)	3.7 (3.0, 4.4)	2.5 (1.8, 3.0)
Transmission										
IDU	21.0 (20.8, 21.1)	17.8 (17.6, 17.9)	15.0 (14.9, 15.2)	12.7 (12.5, 12.9)	10.7 (10.4, 11.0)	8.8 (8.6, 8.9)	7.1 (6.9, 7.2)	5.5 (5.4, 5.6)	4.6 (4.5, 4.7)	2.5 (2.4, 2.5)
Unsafe sexual contact	31.4 (30.3, 32.4)	27.2 (26.2, 28.3)	23.9 (22.8, 24.9)	20.2 (19.1, 21.3)	17.2 (16.1, 18.3)	14.3 (13.2, 15.4)	11.5 (10.4, 12.6)	9.4 (8.5, 10.3)	6.1 (5.3, 6.8)	2.5 (2.4, 2.5)

HIV, human immunodeficiency virus; WHO, World Health Organization; TB, tuberculosis; IDU, injection drug use.

**Table 3.** Life expectancy among HIV-positive patients by province in Iran (1987-2016)

Province	Life expectancy (yr)	95% CI	
		LL	UL
Tehran	31.4	30.2	32.6
Semnan	30.3	17.6	43.0
Qom	28.6	26.3	31.0
Markazi	25.8	24.4	27.3)
Yazd	25.3	21.4	29.2
Mazandaran	25.0	21.5	28.5
Qazvin	24.3	21.7	26.9
Isfahan	24.2	22.3	26.1
Khorasan	23.8	21.8	25.8
Bushehr	23.3	20.8	25.5
Kermanshah	22.9	21.7	24.1
Fars	22.5	21.6	23.4
Kurdistan	21.7	19.5	23.8
Alborz	21.3	19.0	23.7
Hamadan	21.2	19.4	23.0
East Azerbaijan	21.2	19.4	22.9
Hormozgan	20.0	18.3	21.7
Khozestan	19.9	18.6	21.2
Chaharmahal and Bakhtiari	19.9	16.4	23.3
Golestan	19.1	17.7	20.5
Ardebil	19.1	13.3	24.9
Zanjan	18.8	15.6	22.0
West Azerbaijan	17.6	15.9	19.2
Kerman	17.6	14.3	20.8
Lorestan	17.4	16.5	18.3
Sistan and Baluchestan	17.4	14.8	19.8
Qilan	16.8	14.4	19.2
Ilam	14.7	12.6	16.8
Kohgiluyeh and Boyer-Ahmad	13.2	11.6	14.8
National	23.1	22.6	23.5

HIV, human immunodeficiency virus; CI, confidence interval; LL, lower limit; UL, upper limit.
